# Pupillary Dilations of Mice Performing a Vibrotactile Discrimination Task Reflect Task Engagement and Response Confidence

**DOI:** 10.3389/fnbeh.2020.00159

**Published:** 2020-09-03

**Authors:** Dan Alin Ganea, Alexander Bexter, Mathias Günther, Pierre-Marie Gardères, Björn M. Kampa, Florent Haiss

**Affiliations:** ^1^IZKF, Medical Faculty, RWTH Aachen University, Aachen, Germany; ^2^Institute of Neuropathology, RWTH Aachen University, Aachen, Germany; ^3^Department of Ophthalmology, RWTH Aachen University, Aachen, Germany; ^4^Department of Neurophysiology, Institute of Zoology, RWTH Aachen University, Aachen, Germany; ^5^Research Training Group 2416 MultiSenses-MultiScales, RWTH Aachen University, Aachen, Germany; ^6^Unit of Neural Circuits Dynamics and Decision Making, Institut Pasteur, Paris, France; ^7^JARA-BRAIN Institute Molecular Neuroscience and Neuroimaging, Jülich Forschungszentrum, Jülich, Germany

**Keywords:** pupillometry, decision making, somatosensory discrimination, confidence representation, 2-AFC, locomotion, arousal

## Abstract

Pupillometry, the measure of pupil size and reactivity, has been widely used to assess cognitive processes. Changes in pupil size have been shown to correlate with various behavioral states, both externally and internally induced such as locomotion, arousal, cortical state, and decision-making processes. Besides, these pupillary responses have also been linked to the activity of neuromodulatory systems that modulate attention and perception such as the noradrenergic and cholinergic systems. Due to the extent of processes the pupil reflects, we aimed at further resolving pupillary responses in the context of behavioral state and task performance while recording pupillary transients of mice performing a vibrotactile two-alternative forced-choice task (2-AFC). We show that before the presentation of task-relevant information, pre-stimulus, pupil size differentiates between states of disengagement from task performance vs. engagement. Also, when subjects have to attend to task stimuli to attain a reward, post-stimulus, pupillary dilations exhibit a difference between correct and error responses with this difference reflecting an internal decision variable. We hypothesize that this internal decision variable relates to response confidence, the internal perception of the confidence the subject has in its choice. As opposed to this, we show that in a condition of passive performance, when the stimulus has no more task relevance due to reward being provided automatically, pupillary dilations reflect the occurrence of stimulation and reward provision but not decisional variables as under active performance. Our results provide evidence that in addition to reflecting attentiveness under task performance rather than arousal *per se*, pupil dilations also reflect the confidence of the subject in his ensuing response. This confidence coding is overlaid within a more pronounced pupil dilation that reflects post-decision components that are related to the response itself but not to the decision. We also provide evidence as to how different behavioral states, imposed by task demands, modulate what the pupil is reflecting, presumably showing what the underlying cognitive network is coding for.

## Introduction

Pupillometry has been widely used to assess cognitive processes. It has long been known that, when observed under constant light conditions, changes in pupil size reflect underlying brain activity (Loewenfeld, 1958). Seminal studies showed that such changes reflect emotional arousal (Hess and Polt, 1960), cognitive load (Kahneman and Beatty, 1966), and perceptual relevance (Hakerem and Sutton, 1966). Though it is unclear what evolutionary behavioral advantage such dilations might hold (Mathôt, 2018). Changes in pupil size have also been shown to reflect arousal or alertness (Bradley et al., 2008; Vinck et al., 2015), correlate with bouts of locomotion (McGinley et al., 2015; Mineault et al., 2016; Shimaoka et al., 2018) and correlate with synchronized cortical activity (Reimer et al., 2014). Pupil dilations are also a marker of perceptual selection or states of attention switching, indicating as to what underlying cognitive substrate is being perceived (Einhäuser et al., 2008). Also, when human subjects are actively engaged in a task, such changes in pupil size correlate with an increase in mental effort and cognitive load (Hess and Polt, 1964; Kahneman and Beatty, 1966, 1967; Beatty, 1982b) and reflect decision-related processes (Preuschoff et al., 2011; Fiedler and Glöckner, 2012; Kloosterman et al., 2015; de Gee et al., 2017) with the decision-related component shown to hold information regarding the choice that ends the decision process (Einhäuser et al., 2010) but also decision-related information before the decision-related response (de Gee et al., 2014). Pupil size is also indicative of optimal performance (McGinley et al., 2015; Schriver et al., 2018) since it is taken as a proxy of arousal states that modulate cortical activity and signal processing involved in decision making in rodents (Mineault et al., 2016; McGinley et al., 2015) and humans (Murphy et al., 2014b) exhibiting a U-shaped relationship between baseline pupil size and performance levels. This U-shaped relationship has also been proposed for Locus Coeruleus (LC) tonic firing levels (Aston-Jones et al., 1999; Usher et al., 1999). This correlates with tonic and phasic LC activity and the LC-NE theory of adaptive gain (Aston-Jones and Cohen, 2005). A tonic dominated LC state would result in overall small or larger pupil size, unresponsive to task events, while phasic LC state would result in lower baseline pupil size and dilations that reflects task-relevant events (Aston-Jones et al., 1994, 1999; Usher et al., 1999; Clayton et al., 2004). Indeed, pupil dilations under constant illumination are presumably taken as a proxy to LC processing (Aston-Jones and Cohen, 2005; Murphy et al., 2014a; Reimer et al., 2016). Though there is also evidence that links pupillary dilations to Colliculi and Cingulate cortex activity (Joshi et al., 2016). In rodents, the cholinergic system has also been shown to correlate with pupillary dilations (Reimer et al., 2016). Since pupillary responses were shown to occur in response to a variety of behaviors, attention states, and overall cognitive function, we aimed at further resolving pupillary dilations during task-related behavior in mice. As opposed to previous studies in mice that used Go/noGo (Lee and Margolis, 2016) or signal detection (McGinley et al., 2015) tasks a vibrotactile two-alternative forced-choice task (2-AFC) was used in this study. Combining a 2-AFC task, which permits altering task difficulty and temporally separating evidence presentation from task response, together with pupillometry allowed us to track pupillary dilations in the context of specific behavioral states as reflected by the degree of task engagement, and levels of task performance as a function of varying difficulty.

Our results show that when subjects are actively performing the task, arousal levels do not influence performance. Pupillary dilations also differentiate between correct and error task responses. This is hypothesized to reflect a difference in response confidence. Additionally, when temporally separating stimulus from response this correct-error dilation difference is already observed in the post-stimulus, but pre-response phase, hence not emanating from licking related motor activity or reward anticipation. This phase exhibits a slow and reduced dilation that is maintained until task feedback is provided following which a post-response phase starts that exhibits a marked, large and fast, dilation relative to the pre-response component. This second dilation is locked to the response and mainly reflects motor or reward-related components.

## Materials and Methods

### Animals and Surgery

For all experiments, male C57BL/6J mice were used (Charles River). Experiments were approved by North Rhein-Westphalia State Agency for Nature, Environment and Consumer Protection (Landesamt für Natur, Umwelt und Verbraucherschutz Nordrhein-Westfalen, LANUV) and conformed to ethical regulations of German Law for Protection of Animal Welfare. Surgery was conducted to implant mice with a head-fixation bar. Mice were anesthetized with isoflurane in oxygen (3% induction, 1.5% maintenance; V/V) and body temperature was maintained at 37°C with a feedback-controlled heating pad. Analgesia (Buprenorphine; 0.1 mg/kg) was injected S.C. The fur over the skull was removed and the skin was incised using a scalpel. Several drops of a local analgesic agent (Bupivacaine; 0.25%; Actavis, New Jersey, NJ, United States) were used for the incision area. Connective tissue was removed and a bonding agent (DE Healthcare Products) was applied over the bone and polymerized with blue light. Next, blue light polymerizing dental cement (DE Healthcare Products) was used to attach a titanium head bar to the skull. Finally, the skin was sutured around the dental cement cap. An antibacterial ointment (Gentamicin) was applied over the surgery area and antibiotics were added to the drinking water of the animals (Baytril; 25 mg/ml). Animals were monitored and allowed a week of recovery before training commenced, with food and water *ad libitum*. Mice were housed separately and maintained under an inverted 12 h light cycle regime.

### Behavior Procedure and Setup

**Figure 1:**
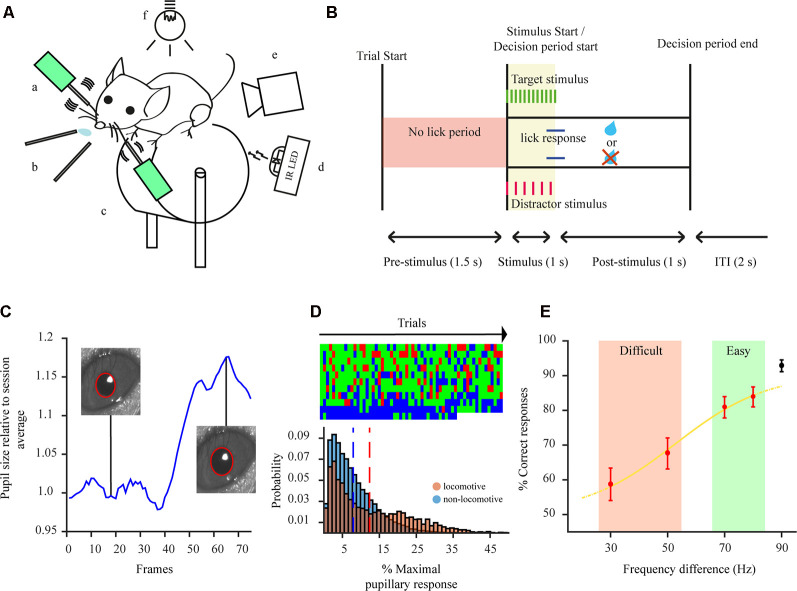
Two-alternative forced choice task (2-AFC) and pupillometry overview. **(A)** Experimental setup: a—whisker stimulators; b—water spouts; c—wheel; d—IR LED illumination; e—pupil tracking camera; f—ambient illumination. **(B)** Schematic of a trial sequence for the 2-AFC task used to test animal behavior.** (C)** Example of a pupillary dilation trace for one behavioral trial. Pupillary trace is shown in blue. *Insets*: examples of pupil detection for two different frames. Recording duration of 2.5 s. **(D)** Top: example of animal responses (correct—green, error—red, miss—blue) during the performance of the 2-AFC task during one session. Each additional row represents 60 consecutive trials within the session; Bottom: distributions of maximal pupil dilation for non-locomotive (blue) and locomotive trials (red), dashed lines show the mean of the distribution. **(E)** Example psychometric response curve for one animal.

Mice were trained to perform a vibrotactile 2-AFC task (Mayrhofer et al., 2013). Briefly, upon commencement of training mice were subjected to a water deprivation regime during weekdays, receiving 1 ml of water per training day and water *ad libitum* during weekends. Weight was monitored daily throughout the water deprivation period. If a loss of over 20% of body weight was observed compared to the non-deprived weekend days, water was supplemented. Mice were handled and acclimatized to the experimenter for 1 week. After acclimatization, mice were head-fixed for increasing periods until accepting 1 ml of water while head fixed. Once mice attained this stage, they were placed in the setup on a wheel to monitor locomotion and behavioral training on the detection task began. In general, for the 2-AFC task mice were required to detect a target stimulus (90 Hz) from two simultaneous bilateral frequencies (for detection distractor was 0 Hz, and later for discrimination 10, 20, 40, or 60 Hz). During the discrimination phase, all stimuli were intermixed. Whisker stimuli consisted of 1 s long repetitive pulses (single-period 120 Hz cosine wave) with a maximum deflection amplitude of 400 μm. Stimulation frequency was modulated by changing inter-pulse time intervals. Target stimulus was randomly delivered to the left or right C1 whisker, with a piezo bending actuator (Johnson Matthey, Royston, UK) amplified by a piezo controller (MDT693A; Thorlabs, NJ, USA) with mice having to report the side the target was presented on by licking one of two corresponding left or right water spouts placed in front of them (Figure 1A). Lick detection was conducted by capacitive water spouts connected to an Arduino platform (Arduino UNO Rev3; Arduino, Italy). Water delivery was controlled by solenoid valves (Bürkert, Ingelfingen, Germany). Responses to the task were classified under four categories: correct response with mice being rewarded a water drop delivered through the corresponding spout, error response where no water was rewarded, miss when the animal did not respond with a lick within the decision period window, with no water being rewarded, or a double-lick when mice licked both spouts within a 60 ms period with no water being rewarded. The temporal structure of each trial consisted of a 1-s stimulus presented 1.5 s following trials start, with a response window of 2 s after stimulus initiation. The inter-trial interval was set as 2 s after the response of the animal or end of decision period with a 50% maximal temporal jitter (Figure 1B). Once mice attained performance of 85% correct responses per session in the detection task, discrimination training commenced. For the *delayed response detection task* waterspout movement was controlled by servo-electric motors (Savöx, Taiwan), following a determined delayed period after stimulus initiation (1,000, 1,500, 2,000 ms). This task variant consisted only of detection of the 90 Hz target stimulus. For the *passive engagement task*, highly trained mice (>90% correct responses for detection task) were provided only with a target whisker stimulation (90 Hz), coupled with the automated provision of the reward or in an additional set of experiments provided only with a reward. Control of the behavioral sessions and behavioral data analysis was conducted with custom written LabVIEW (National Instruments, RRID:SCR_014325) and MATLAB software (MathWorks, RRID:SCR_001622).

### Locomotion

To monitor for locomotion, mice were placed on a Polystyrene (Styrodur^®^) wheel, 20 cm diameter, and movement was tracked using an optical incremental encoder (Optical miniature encoder 2400; Kübler, Germany). Locomotion was determined as any movement crossing a >5 cm/s threshold during the duration of the trial.

### Pupil Imaging and Detection

Images were acquired using a Point Grey Chameleon3 camera (Point Grey Research) at 30 FPS with a 50 mm lens and the pupil illuminated by an Infrared light-emitting diode (led). Throughout the behavioral session, the setup was maintained under constant white light illumination, with the pupil in a dynamic range. Pupil movies were recorded separately for each trial (15 frames for baseline). For image acquisition, a custom-written LabVIEW software (National Instruments, RRID:SCR_014325) was used and pupil detection and fitting was conducted offline with custom-written MATLAB software (MathWorks, RRID:SCR_001622). For pupil detection, a threshold was determined for each frame and the image converted to a binary image. The pupil was detected using a circle fitting algorithm that detects the mean [*x*, *y*] coordinates of the pupil in the binary image. For determining the validity of the detection, 20% random frames in each movie were visually analyzed by the experimenter. The validity criterion was set as >98% fit for all non-blinking frames per session. As blinking results in a quick and sudden change in measured pupil size, a threshold for the differential of the pupil transient was used and trials, where blinking was detected, were removed from all subsequent analysis.

### Data Analysis

#### Behavioral Data Analysis

Psychophysical response curves for each animal were analyzed with a MATLAB toolbox for psychophysical data analysis (psignifit version 2.5.6; see http://bootstrap-software.org/psignifit), which implements a maximum-likelihood method (Wichmann and Hill, 2001). We used a logistic function:

$$\begin{array}{c} \psi (x,\alpha ,\beta ,\gamma ,\lambda )=\gamma +\left(1-\gamma -\lambda \right)F(x;\alpha ,\beta ),\\ F(x;\alpha ,\beta )=1/\left(1+\exp\left[\frac{\alpha -x}{\beta }\right]\right) \end{array}$$

to fit the data points (parameters: *α, β, γ* = 0.5, λ [0 0.2]) and obtain the inflection point of the discrimination threshold and slopes. Confidence intervals to the response for each stimulus pair were computed based on a binomial distribution with a confidence level of 95%. Performance in the 2-AFC task was computed as:

$$\%\text{Performance}=\frac{\text{correct responses}}{\text{correct responses}+\text{error responses}}\times 100$$

Miss categorization for attentive and non-attentive periods during the session was conducted using a cut-off criterion where 50% of trials within a 10-trial window consisted of miss. Miss trials before this cut-off were categorized as being during an attentive period and miss trials following this categorized as being during a non-attentive period.

All data used in this study consists of mice having a performance above 85% correct responses per session in the detection task.

#### Pupil Data Analysis

For comparing pupil dilations between animals and sessions, pupil diameter per data point in each session was divided by the average pupil size of that session, i.e., normalized pupil size. For determining the pupillary dilation transient per trial (change relative to pre-stimulus period), for each trial, the average pre-stimulus, normalized pupil size was calculated and subtracted from each normalized pupil size sample point, with the result divided by the average value of the pre-stimulus normalized pupil size. For quantifying the pupillary response, the maximal pupil dilation per trial was used, resulting in the maximal pupillary response.

#### Experimental Design and Statistical Analysis

**Figure 2:**
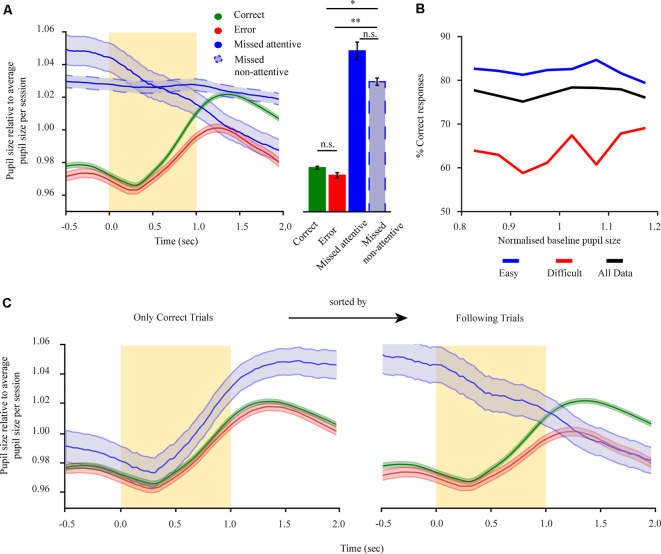
Pre-stimulus pupillary size of mice performing a 2-AFC task reflects task engagement state and holds no information regarding subsequent performance. **(A)** Left: mean pupil size for all trials separated into correct trials (green), error trials (red), miss trials (solid-blue), and miss trials during the non-attentive period at the end of the session (dashed-blue). Right: mean pupil size for the pre-stimulus period. **(B)** Performance for all animals (*n* = 8) in dependence of baseline pupil size. Performance is shown for three groups: all trials (black), difficult trials (distractor > 30 Hz; red), and easy trials (distractor ≤30 Hz; blue).** (C)** Dilation transients showing the history dependency of the pupil size using the example of rewarded trials and trials following rewarded trials. Left: only rewarded (correct) trials are shown. The trials are separated by the outcome of the following trial. Right: trials following rewarded (correct) trials (the same results were seen when restricting the analysis to miss following error trials). The yellow rectangle represents stimulus. *p < 0.05, **p < 0.01, n.s.: not significant.

**Figure 3:**
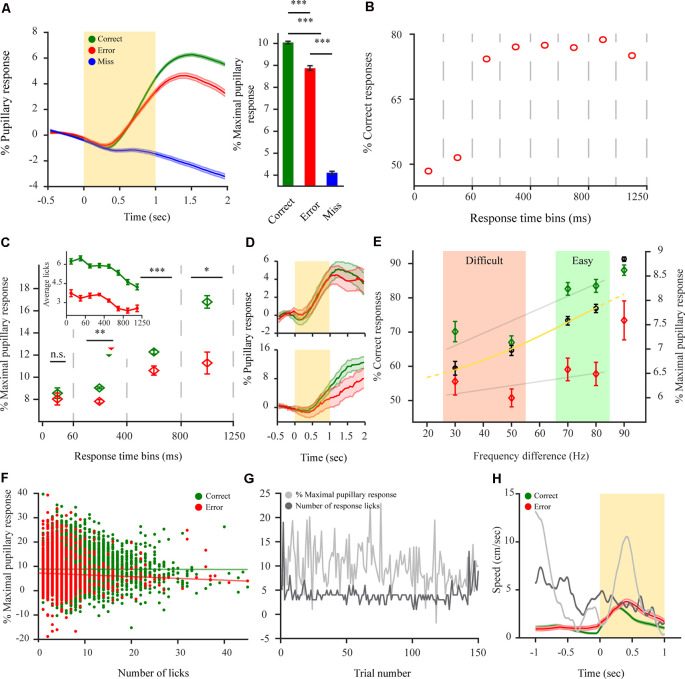
Pupillary dilations of mice performing a 2-AFC task differ depending on animal response and reflect internal decision components. **(A)** Left: pupil dilation transients for mice performing a 2-AFC task (*n* = 8 mice; *N* = 92 sessions) for correct (green), error (red) and miss (blue) responses. Right: pupil response magnitude following stimulus onset for the different response types. **(B)** %Correct responses for different time bins. Dashed vertical lines represent time bins. **(C)** Pupil response magnitude for different response time (RT) time bins for correct and error responses. Dashed vertical lines represent time bins used for averaging pupil response magnitude. Median response time represented by triangles for correct (green—264 ms) and error (red − 260 ms) responses. *Inset*: the average number of licks per trial for correct and error trials for different time bins as in panel **(B)**. **(D)** Example pupillary dilation traces for correct and error responses in two response time bins. Top: 0 to 60 ms RT bin. Bottom: 800 to 1250 ms RT bin. **(E)** Decrease in pupil response magnitude correlates with a decrease in animal performance as difficulty increases for performance in a 2-AFC discrimination task for correct responses but not for error responses. Black circles are average performance across mice with the logistic fit (yellow). Gray lines represent linear fit for the discrimination task. **(F)** Scatterplot of pupil response magnitude and the corresponding number of licks for all trials (12,827 correct (green), 4,056 error (red) trials). Straight lines represent linear fit for the data points. **(G)** Example of first 150 correct trials (non-consecutive) showing no correlation between maximal pupil dilation and the number of corresponding licks per each trial. **(H)** Sub-threshold average locomotion speed for correct and error trials. The yellow rectangle represents stimulus. Gray lines are two examples of over-threshold locomotive trials. Over-threshold locomotive trials were exempt from analysis and are shown only as a relative example (see “Materials and Methods” section). *p < 0.05, **p < 0.01, ***p < 0.001, n.s.: not significant.

**Figure 4:**
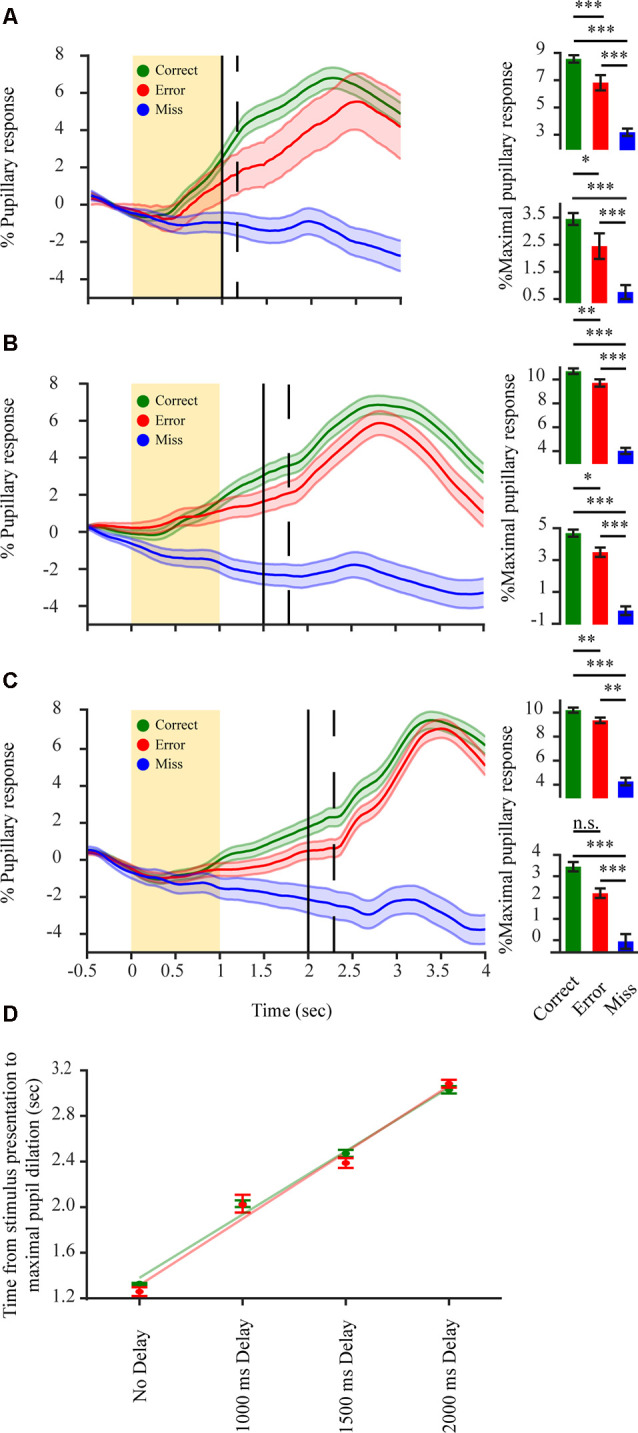
Pupillary dilations in a delayed 2-AFC detection task reflect decision variables but mainly encode for post-decision components. **(A–C)** *Left in each panel*: average pupillary dilation transients for correct (green) error (red) and miss (blue) responses in a delayed response detection task (*n* = 3 mice). Spout delay from stimulus onset 1,000 ms (seven sessions); 1,500 ms (eight sessions); 2,000 ms (nine sessions); respectively. The yellow rectangle represents stimulus; Black vertical line represents water spouts presentation; dashed vertical line represents mean RT. *Bottom right in* *each panel*: average percent maximal pupillary response in a period of 500 ms before water spout presentation. *Top right in each panel*: average percent maximal pupillary response in the period following water spout presentation. **(D)** Time difference between stimulus onset and maximal pupillary response increases as a correlation of the delay period for correct (green) and error (red) responses. Green and red lines represent linear fit for correct and error responses respectively. *p < 0.05, **p < 0.01, ***p < 0.001, n.s.: not significant.

**Figure 5:**
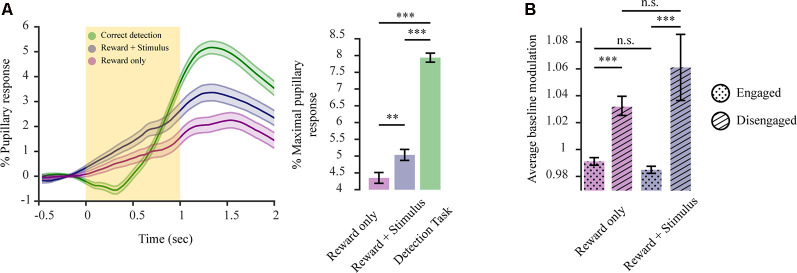
Pupillary transients for a state of passive performance hold perceptual information for both stimulus and reward, reflecting a state of quasi-engagement. **(A)** Pupil dilation transient differs for states of active performance vs. passive performance Left: average dilation transients (*n* = 3 mice) for reward only (purple; 2,246 trials), reward + stimulus (blue; 2,069 trials) and correct responses in a detection task (green; 2,419 trials). Right: average pupil response magnitude for the different behavioral conditions. **(B)** Average pupil size during the baseline period (before stimulus onset) for the passive behavior states and their corresponding disengagement periods. The yellow rectangle represents stimulus. **p < 0.01, ***p < 0.001, n.s.: not significant.

The experimental design for baseline period analysis (Figure 2) and post-stimulus analysis (Figure 3) consisted of eight mice. The delayed response task (Figure 4) consisted of three mice and a passive engagement task (Figure 5) consisted of three mice. For determining significance between the different conditions in the baseline period, pupil size was averaged per trial for the pre-stimulus period, referred to as average pupil size modulation, and repeated measures one-way ANOVA used followed by paired contrasts as appropriate. For determining significance between conditions of the post-stimulus pupillary response, the maximal pupil size following stimulus onset was used as a test variable, and this maximal pupillary response analyzed using a repeated measure one-way ANOVA followed by paired contrasts as appropriate. For correlations, we used a one-sided Kendall rank coefficient for the test statistic, and a linear fit of the data was applied. Unless stated otherwise shaded error bars for the pupillary dilation transient represent 95% Confidence Interval (95CI) and error bars represent Standard Error of the Mean (SEM). Data in the text is presented as mean ± SEM. Statistical analysis was conducted using MATLAB software (MathWorks, RRID:SCR_001622).

## Results

The pupil of mice was tracked while performing a 2-AFC task (Figures 1A,B). Mice performed on average 336.2 ± 8.4 trials per session. Pupil size fluctuated throughout the session and through each trial (Figure 1C). It is known that locomotion correlates with an increase in pupil size and might reflect a state of hyperarousal (McGinley et al., 2015; Vinck et al., 2015; Mineault et al., 2016). Indeed, pupillary responses were dominated by larger dilations when mice were locomotive (Figure 1D-bottom). This might mask other effects arising from task performance. As such, we excluded locomotive trials from the analysis. In 2-AFC tasks, there are three possible behavioral response types: correct, error, and miss (no response). We categorize correct and error responses as a task engaged state and miss trials as indicative of a task disengaged state. Miss category could also be further separated into sparse miss trials occurring during periods of engagement (attentive period) or as a batch at the end of the session (non-attentive period). Mice were highly engaged in the task, represented by their consistent response to presented stimuli throughout each session (84.1 ± 4.9% of all trials; Figure 1D-top). Employing a 2-AFC task also permits to separate performance, based on psychophysical measures of the subject’s percept, into easy and difficult task categories (Figure 1E) which were further used to categorize pupil dilations.

### Pre-stimulus Pupillary Size of Mice Performing a 2-AFC Task Reflects Task Engagement State and Holds No Information Regarding Subsequent Performance

To relate pupil dilations with task variables, we focused on pupil dilations in pre- and post-stimulus periods, as these revolve around the moment of task-related evidence presentation. Pupil size before stimulus onset (baseline period) was previously related to arousal levels (Gilzenrat et al., 2010) and optimal performance when related to task behaviors (McGinley et al., 2015). However, these results mainly stem from Go/noGo tasks which might involve different cognitive functions to the more demanding 2-AFC task. As such, we wanted to examine how baseline pupil size relates to task performance in the current paradigm. Baseline pupil size was significantly different for the four response conditions (Figure 2A; *F*_(3,21)_ = 2.17, *p* < 0.01), with baseline pupil size during states of task disengagement being larger than when in engaged states (*F*_(1,7) correct non-attentive miss_ = 3.93, *p* < 0.001; *F*_(1,7) error non-attentive miss_ = 2.23, *p* < 0.05). No difference in pupil size for correct and error responses was observed (*F*_(1,7)_ = 0.60, *p* = 0.43; *M*_(correct)_ = 0.977 ± 0.001; *M*_(error)_ = 0.972 ± 0.002). While we did observe a significant difference for attentive and non-attentive miss conditions (*F*_(1,7)_ = 0.95, *p* = 0.45; *M*_(attentive miss)_ = 1.048 ± 0.001; *M*_(non-attentive miss)_ = 1.029 ± 0.002) this arises due to the history dependence from previously engaged trials during the attentive period (Figure 2C) with both miss types still evidently different to engaged responses. Hence, baseline pupil size is indicative of the engagement state. To test whether the baseline period also holds information regarding task performance, we analyzed the performance of mice with respect to baseline pupil size for engaged trials (correct and error; Figure 2B). This revealed that task performance is not correlated with baseline pupil size (Figure 2B, black solid line; *r*_τ_ = 0.07, *p* = 0.64). Furthermore, we tested if baseline pupil size and performance exhibit a different relationship depending on task difficulty as hyper- or under arousal might influence performance. However, no dependency of baseline pupil size over performance for different task difficulties were revealed (Figure 2B, blue and red; *r*_τ easy_ = 0.21, *r*_τ hard_ = 0.36, *p* = 0.14), with performance levels dropping overall per difficulty level but remaining constant as a function of baseline pupil size. As such, baseline pupil size seems to hold no perceptual information regarding task performance. For mice performing a 2-AFC task baseline pupil size, if taken as a proxy of arousal levels, has no effect over task performance manifesting only overall engagement or disengagement states. Due to the relatively low occurrence of attentive miss responses and the significant difference to engaged responses both types of misses were pulled under a miss condition in further analysis.

### Post-stimulus Dilation Transients of Mice Performing a 2-AFC Task Differ Depending on Animal Response Reflecting Internal Decision Components

Next, to distinguish perceptually related task responses as reflected by the pupil, pupillary dilation transients were baselined relative to the pre-stimulus period, resulting in a pupillary dilation transient that reflects the perceptual content of the information withheld by the pupil (i.e., relative to stimulus onset and evidence accumulation). A significant difference was observed between pupillary dilation transients for the three different response types to the task (Figure 3A; *F*_(2,14)_ = 397.1, *p* < 0.001). Pupil dilation transients during the disengaged state remained principally unresponsive to stimulation and revealed only a late (~700 ms) and small pupillary response (*M*_(miss) =_ 4.06 ± 0.08). This was significantly different from the pupillary response for both correct (*F*_(1,7)_ = 1283.3, *p* < 0.001), and error (*F*_(1,7)_ = 470.58, *p* < 0.001). Contrary to this, pupillary dilation transients during the engaged state, revealed a faster (~330 ms) and increased response following stimulation, for both correct (*M*_(correct)_ = 9.96 ± 0.05) and error (*M*_(error)_ = 8.79 ± 0.09) with correct responses showing a significantly larger pupillary response magnitude than error responses (Figure 3A; *F*_(1,7)_ = 17.56, *p* < 0.001). This difference between the pupillary dilation transients for correct and error responses might on the one hand arise due to coding of externally induced signals such as reward attainment for correct responses but also, on the other hand from various internal decision components. Hence, we were interested in characterizing this difference and understanding where it emanates from. First, to understand the relationship between pupil dilation and decision components, we analyzed response time (RT) as it is indicative of the decision being stimulus-based or merely impulsive (non-stimulus based) with very early RTs implying a guessing strategy (Carpenter and Williams, 1995). Indeed, when analyzing task performance as a function RT for different response time bins (Figure 3B), for early bins of RTs (<60 ms) performance is at chance levels. Indicating that RT can be used as a measure for categorizing responses between evidence-based and impulsive ones. Hence, we wanted to observe whether the pupillary response also diverges between correct and error responses as a function of when the RT is provided, by observing the pupillary response magnitude occurring for different RT bins. This analysis revealed that pupillary dilations indeed exhibited a different phenotype when task responses occur impulsively as opposed to evidence-based. This was manifested as there being no difference between the pupillary response magnitude of correct and error responses for these early responses (see example in Figure 3D-top), when provided in the first 60 ms (*F*_(1,7)_ = 0.11, *p* = 0.74; *M*_(correct, 0–60 ms)_ = 8.51 ± 0.33; *M*_(error, 0–60 ms)_ = 8.09 ± 0.38). But for later RT bins, where the choice is guided by the stimulus (i.e performance above chance levels; 60–1,250 ms; see example in Figure 3D-bottom) the pupillary response magnitude exhibited a difference and was larger for correct responses than for error throughout all bins (*F*_(1,7)_ = 10.59, *p* < 0.01; *M*_(correct, 60–400 ms)_ = 8.74 ± 0.06; *M*_(error, 60–400 ms)_ = 7.51 ± 0.12; *F*_(1,7)_ = 14.02, *p* < 0.001; *M*_(correct, 400–800 ms)_ = 12.12 ± 0.16; *M*_(error, 400–800 ms)_ = 10.60 ± 0.34; *F*_(1,7)_ = 5.19, *p* < 0.05; *M*_(correct, 800–1250 ms)_ = 16.38 ± 0.44; *M*_(error, 800–1250 ms)_ = 11.12 ± 0.79). This differentiation between early RTs, and late RT time bins, indicates that the correct-error pupil dilation difference cannot be related to the attainment of the reward itself as task structure is constant regardless of when the response is provided. Despite the difference not being reward-attainment related *per se*, there exists the possibility that the observed correct-error difference is driven by aspects of motor activity and not decision variables (i.e., licking or locomotive aspects). To examine this possibility, we analyzed the mean number of licks elicited for correct or error responses for different time bins, to observe whether licking behavior follows the same phenotype as the divergent pupil dilations for impulsive vs. evidence-based responses. The analysis revealed this is not the case, as more licks are presented for correct responses also during the early time bins where there is no divergent pupil dilation as a function of the response (Figure 3C-inset) indicating that licking cannot in itself explain the correct-error difference. To further control for this, we directly analyzed the magnitude of the dilations and the number of licks elicited (Figure 3G). Indeed, no relation was observed between them with no correlation between the maximal pupil response and the number of licks elicited for both correct and error responses (Figure 3F; *r*_r correct_ = −0.26, *p* = 0.07; *r*_r error_ = −0.09, *p* = 0.57). Pupil dilating more for correct responses regardless of the number of licks. Finally, the correct-error pupil dilation difference also cannot be explained by locomotion profile, as mice are more locomotive during error trials despite smaller pupil dilations (Figure 3H). Importantly, when all these findings are taken together, they reveal that the correct-error difference between pupillary dilation transients cannot arise due to external factors such as differences in reward attainment, locomotion or licking between correct and error trials, but rather, as indicated by the early time bins result, suggesting it reflects internal decision variables. This notion is further supported by the relationship between pupil dilations and task difficulty effects (Figure 3E). As the difference between the target stimulus and distractor stimulus decreases, it becomes more difficult to discriminate between the two simultaneously presented stimuli and solve the task (Figure 3E). With performance being highest for the detection task (*M* = 91.3 ± 0.6% correct responses) and dropping with increased task difficulty, reaching near chance levels (*M* = 59.4 ± 2.0% correct responses). This decrease in performance correlated with a decrease in the pupillary response magnitude for correct responses but not with error responses which exhibited no significant decrease as a function of difficulty level for the discrimination task (Figure 3E; *r*_τ correct_ = 0.388, *p* < 0.001; *r*_τ error_ = 0.003, *p* = 0.402). Hence, as performance drops to chance levels the pupillary response magnitude for correct and error trials tends to converge, indicating again that when the choice is random pupillary dilation becomes similar. Taken together, this further suggests that the correct-error pupil dilation reflects an internal decision variable.

### Pupillary Transients in a Delayed Response 2-AFC Detection Task Reflect Decision Variables but Mainly Encode for Post-decision Components

If indeed the correct-error difference in perceptually related pupil dilations is related to decision variables, it might also be manifested following evidence accumulation but prior to response feedback. Due to the slow kinetics of the pupillary response, it is possible that any decision related response reflected by the pupil in the period between presentation of the stimulus and RT would not be observed due to task design (temporal separation between stimulus and RT). In order to determine if such pupillary decisional representations do occur during this period, we postponed the motor output by training the mice to perform a delayed response detection task with the water spouts only being presented after a delay period following stimulus onset. Pupillary dilation traces for both correct and error responses began increasing following stimulation (Figures 4A–C), showing a significant difference between the pupillary dilation trace for correct, error and miss responses already before the animal provided its response to the task or received any response feedback (*F*_(2,4) 1,000 ms_ = 82.81, *p* < 0.001; *F*_(2,4) 1,500 ms_ = 53.46, *p* < 0.001; *F*_(2,4) 2,000 ms_ = 26.42, *p* < 0.001). This increased pupillary dilation trace for correct and error responses was maintained throughout the stimulus—RT interval (1,000 ms: *F*_(1,2) correct-error_ = 30.08, *p* < 0.001, *F*_(1,2) correct-miss_ = 158.08, *p* < 0.001, *F*_(1,2) error-miss_ = 53.05, *p* < 0.001 *M*_correct_ = 3.45 ± 0.22; *M*_error_ = 2.45 ± 0.47; *M*_miss_ = 0.76 ± 0.25; 1,500 ms: *F*_(1,2) correct-error_ = 10.76, *p* < 0.01, *F*_(1,2) correct-miss_ = 90.73, *p* < 0.001, *F*_(1,2) error-miss_ = 76.85, *p* < 0.001 *M*_correct_ = 4.69 ± 0.22; *M*_error_ = 3.49 ± 0.29; *M*_miss_ = −0.18 ± 0.27; 2,000 ms: *F*_(1,2) correct-error_ = 17.96, *p* < 0.01, *F*_(1,2) correct-miss_ = 54.72, *p* < 0.001, *F*_(1,2) error-miss_ = 10.31, *p* < 0.01 *M*_correct_ = 3.45 ± 0.22; *M*_error_ = 2.20 ± 0.22; *M*_miss_ = −0.06 ± 0.35). This again excludes that the correct-error difference is due to reward attainment. Following the RT of the animal, pupillary responses exhibited a second and more pronounced, significant increase in pupillary dilation (*F*_(2,4) 1,000 ms_ = 236.16, *p* < 0.001; *F*_(2,4) 1,500 ms_ = 123.34, *p* < 0.001; *F*_(2,4) 2,000 ms_ = 97.72, *p* < 0.001) with correct trials still showing the largest pupil dilation and miss trials hardly any difference (1,000 ms: *F*_(1,2) correct-error_ = 4.51, *p* < 0.05, *F*_(1,2) correct-miss_ = 452.70, *p* < 0.001, *F*_(1,2) error-miss_ = 300.84, *p* < 0.001, *M*_correct_ = 8.56 ± 0.27; *M*_error_ = 6.82 ± 0.56; *M*_miss_ = 3.18 ± 0.27; 1,500 ms: *F*_(1,2) correct-error_ = 4.95, *p* < 0.05, *F*_(1,2) correct-miss_ = 223.96, *p* < 0.001, *F*_(1,2) error-miss_ = 176.17, *p* < 0.001, *M*_correct_ = 10.69 ± 0.23; *M*_error_ = 9.71 ± 0.31; *M*_miss_ = 4.02 ± 0.25; 2,000 ms: *F*_(1,2) correct-error_ = 1.37, *p* = 0.24, *F*_(1,2) correct-miss_ = 176.57, *p* < 0.001, *F*_(1,2) error-miss_ = 130.47, *p* < 0.001 *M*_correct_ = 10.20 ± 0.22; *M*_error_ = 9.35 ± 0.23; *M*_miss_ = 4.25 ± 0.32). Average pupillary dilation trace for the disengaged state remained overall unchanged as in the un-delayed task, indicating the unresponsiveness of the pupil when mice become disengaged. For the engaged state, across all delay periods, the increase in delay and the pupillary response magnitude exhibited a positive correlation for both correct and error responses (Figure 4D; *r*_τ correct_ = 0.541, *p* < 0.001; *r*_τ error_ = 0.436, *p* < 0.001). Indicating that the maximal pupillary dilation follows the RT and not the stimulation. Hence, the difference in pupillary dilation transients observed for correct and error responses in the stimulus-RT interval (Figures 4A–C) reflects a stimulus-based decision variable before RT with a second component due to motor output or possible reward attainment following RT.

### Pupillary Transients of Mice in a State of Passive Engagement Hold Perceptual Information for Both Stimulus and Reward and Reflect a State of Quasi-engagement

If the pupil exhibits this decisional variable, how does this manifest itself when the requirement to decide is annulled? To better determine this, we conducted two additional sets of experiments. In the first type of experiment, mice already trained in the 2-AFC task were provided automatically with a water reward upon whisker stimulation (90 vs. 0 Hz) in all trials for several sessions. In the second type of experiment, the same mice were now only provided with a water reward, without whisker stimulation, for several sessions, to observe how decisional coding is altered and whether there is a pupillary representation of the stimulation in addition to the reward. For both experiments, the temporal sequence of the task was the same as in Figure 1B. Hence, in both cases mice were passively performing the task, meaning they were responding to the presented reward without the requirement to solve a task to obtain it. With whisker stimulation losing its task-relevant meaning. When comparing the passive performance states for these mice with their correct responses as provided in the active performance state (as all three conditions contain the attainment of reward; as in Figure 3A) we observed a significant difference in pupillary response magnitudes between both passive performance states and active performance (Figure 5A; *F*_(2,4)_ = 102.86, *p* < 0.001). Under passive performance, the presentation of the *stimulus + reward* vs. *reward only* also elicited a significantly higher pupillary response magnitude (*F*_(1,2) reward vs. reward + stimulus_ = 15.48, *p* < 0.01; *M*_(reward)_ = 4.35 ± 0.16; *M*_(reward + stimulus)_ = 5.03 ± 0.16). This indicates that both stimulus and reward *per se* are encoded additively by the pupillary response. However, our results also reveal that the pupillary response magnitude is higher when mice are actively performing the task vs. both states of passive behaviors (*M*_(active engagement)_ = 7.94 ± 0.13; *F*_(1,2) active engagement vs. reward_ = 172.07, *p* < 0.001; *F*_(1,2) active engagement vs. stimulus + reward_ = 131.21, *p* < 0.001). This reveals the added perceptual coding reflected by the pupil through the necessity to solve the task. It is possible that during passive behaviors mice are in a different state of arousal or attentiveness as cognitive processing required for reward attainment is less demanding. For this scope, we examined baseline pupil size for the various behavioral states relative to its size during the disengagement state (miss responses). For passive performance states, there was a significant difference between reward responsive trials and miss trials (Figure 5B; *F*_(3,6)_ = 4.85, *p* < 0.001), with baseline pupillary size being increased when mice were in a state of disengagement vs. reward responsiveness (engagement)*, stimulus + reward* condition (*F*_(1,2)_ *t*_0.001_; *M*_engaged stimulus + reward_ = 0.985 ± 0.003; *M*_disengaged stimulus + reward_ = 1.06 ± 0.02) and *reward only* condition (*F*_(1,2)_ = 5.59, *p* < 0.001; *M*_engaged reward_ = 0.991 ± 0.003; *M*_disengaged reward_ = 1.032 ± 0.007). No significant difference was observed when comparing miss responses or reward responsive states for the two task variants (*F*_(1,2)_ = 1.09, *p* = 0.29, *F*_(1,2)_ = 0.04, *p* = 0.85). This indicates that despite no requirement of solving a task, during cognitively less demanding behaviors, states of engagement are still different to complete task disengagement. Overall, when actively performing a task, dilations are representing the task variables but also decisional variables arising from the requirement to solve it. This indicates that the pupil reflects varying behavioral requirements imposed on the animal due to different contingencies.

## Discussion

Under conditions of task engagement, pupil dilations in humans were shown to represent various aspects of underlying cognitive activity, among which prediction error (Preuschoff et al., 2011; Braem et al., 2015; Urai et al., 2017), reward anticipation (Chiew and Braver, 2013), required effort or vigor (Zénon et al., 2016) and response confidence (Lempert et al., 2015). Studies using mice might provide an advantage in linking the underlying mechanisms of pupil dilations with neuronal activity, though it remains unclear to what extent pupil dilations in mice represent complex aspects of cognitive function as in humans. In the current study, we show that the pupil is a complex readout system indicative of multiple perceptual phenotypes. When in a state of engagement, defined as task responsiveness, large dilations are observed following task-related evidence presentation (stimulation), with larger dilations for correct than error responses (Figure 3A). We were interested in defining the underlying drive for this pupillary representation. This difference might arise due to external representations such as reward (Lee and Margolis, 2016) or motor-related activity either overt or covert (Richer et al., 1983; Mineault et al., 2016). Our analysis indicates that this difference is not related to such external occurrences. This is exhibited by several factors. There is a lack of correct-error differences in pupil dilation when performance is at chance levels (Figures 3B,C) emanating from an impulsive, non-evidence-based, behavior (Carpenter and Williams, 1995; Mayrhofer et al., 2013). Additionally, but contrary to other studies (though see Lu et al., 2020), in the current study increases in dilation do not correlate with increased licking (Figure 3G) and the increased locomotion during error trials cannot account for increased dilations for correct responses (Figure 3H). Thus, the correct-error dilation difference does not originate from motor reflected activity emanating from licking or locomotive behavior. Furthermore, a reward-driven difference would imply a dilatory difference that is not RT dependent, ruling out also reward as the source of the correct-error difference. Taken together, this supports the notion that the dilatory difference emanates from an internal decision component. Any coding for a decision component should be represented in the post-stimulus and pre-RT period. To test for this, and strengthen our hypothesis, we conducted a delayed-response task that temporally separates decision components from motor responses. Two distinct dilation periods were observed (Figures 4A–C). A slow dilation following stimulation, and a second, more pronounced dilation after response. This slow pre-reward attainment component cannot be explained by licking behavior as it was our experience that for these highly trained subjects hardly any licking occurred before the presentation of the water spouts. While the slow increase in pupil size might represent a latent component of ramping reward anticipation. As anticipation in itself cannot explain the correct-error divergence in pupil size as anticipation *per se* should be modulated by some decisional weight. These findings further support possible decision-related effects, as transients already increased and differed in the first, pre-feedback period exhibiting the correct-error difference, without any external input to drive this increase or divergence. The second dilation, exhibited following RT, is more likely relating to motor responses or the reward itself.

Many decisional components might explain this difference. For instance, many pupil studies in humans find a reward prediction error representation (RPE). Our results are not in line with this notion. RPE would imply increased dilations for errors and a correct-error difference also for early RTs since non-evidence-based prediction should be the same (guessing), while reward outcome differs. Potentially, the difference could also emanate from the underlying response vigor or effort (Zénon et al., 2016). i.e., the willingness of the subject to “invest” more mental effort to attain a reward in the face of difficulty. Since in this study mice are experts in the task, this could occur due to prior knowledge of lower reward probability outcomes for more difficult trials. Alternatively, the pupil might also reflect the actual “mental effort” required to solve more difficult trials. Effort coding has been related to dopamine, with more vigor related to lower dopamine release (Kurniawan et al., 2011; Walton and Bouret, 2019). Dopamine release in turn might be reflected by pupil dilations (de Gee et al., 2017). Our presented data and analysis do not support vigor coding by the pupil as, under the first interpretation, we would have observed larger pupil dilations for more effort requiring trials (Figure 3E). While under the second assumption, the overall drop in pupil dilations for more difficult trials (Figure 3E) could be explained by effort coding but it cannot explain the correct-error difference itself as this is manifested irrelevant to task difficulty (Figures 3E, 4A–C).

Where does this correct-error difference arise from? We hypothesize that our results could be interpreted in terms of representation of response confidence, a perception of how confident subjects are in their ensuing response. First, confidence coding can explain early RT results, as response confidence would be equal or irrelevant when responses are random and not evidence-based. Second, the correct-error difference is observed only when responses are stimulus-based, with dilation transients continuing to increase with increased RT (Figures 3C, 4). It would be appropriate for a notion like response confidence to be maintained within an underlying network until RT, awaiting choice feedback. For highly trained animals, responses leading to a correct outcome would be presumed to be accompanied by higher choice confidence due to prior experience. Third, response confidence can also explain the differences in dilations with respect to task difficulty. Higher response confidence being exhibited as larger dilation for easier tasks but dropping with increased difficulty and the correct-error difference ultimately converging at chance level performance when based on guessing (Figure 3E). Fourth, in support of confidence coding, a recent study (Lak et al., 2017) linked response confidence with the dopaminergic system. Indeed, LC modulates dopaminergic activity in both Ventral Tegmental Area (VTA) and Substantia Nigra (Grenhoff et al., 1993; Zhu, 2018) and VTA afferents innervate LC (Ornstein et al., 1987). While this study did not address pupil dilations it is conceivable that the dopaminergic system reflects confidence through dilations either driven by LC activation of dopaminergic loci or prefrontal cortex feedback arising from these interconnected systems (Arnsten and Goldman-Rakic, 1984; Sara and Hervé-Minvielle, 1995; Jodo et al., 1998). When taken together these results support our hypothesis that the decisional variable observed through the correct-error dilation difference relates to response confidence.

Pupil studies in mice, mainly studied pupil size in correlation with arousal levels (Murphy et al., 2014a; Reimer et al., 2014) indicated through locomotion (Mineault et al., 2016; Shimaoka et al., 2018) or surprise (Vinck et al., 2015). Further, arousal levels influence performance (McGinley et al., 2015; Schriver et al., 2018) manifested as a U-shaped relationship between the two (Murphy et al., 2011). Though, see Kahneman and Beatty (1967); Beatty (1982a); Karatekin et al. (2007) and Neske et al. (2019). In the current study, under task performance, baseline pupil size is not directly an arousal marker but rather indicates engagement. Overall, when subjects are disengaged, pre-stimulus pupil size is not overtly coding task-relevant information (Figure 2A) and not relating to performance levels for more difficult trials (Figure 2B). This shows that for the current task if baseline pupil size is taken as a proxy of arousal, arousal levels do not affect performance. This is in discrepancy with previous results (McGinley et al., 2015). This could be explained by different task modalities being used (McGinley et al., 2015; Neske et al., 2019), different cognitive requirements imposed by the 2-AFC task and Go/noGo or detection tasks response categorization, where perceptual failure or lack of motivation are less distinguishable or lighting conditions in the experiment. Under low light conditions used in previous studies, pupil size might be mainly influenced by sympathetic input. However, parasympathetic input would dominate in the ambient light condition used in the present study (Steinhauer et al., 2004). Hence, we conclude that under the described task conditions baseline pupil size, holds no information for perceptually relevant task processing. Pre-stimulus pupil size reflects engagement or disengagement states, not a general state of arousal.

The behavioral state or task demands, based on what various, underlying neuronal mechanisms are directed towards, may well influence pupil diameter. As such, the stimulus-reward association was altered by decoupling the stimulus detection requirement from reward attainment. A condition we refer to as passive performance. Under these conditions, dilations were smaller compared to correct responses under active performance, even though stimulus and reward presentation were the same (Figure 5A). Under passive performance, when both reward and stimulation were presented, dilation was increased compared to when only the reward was presented, indicating that under passive performance both reward and stimulus are reflected by the pupil. Thus, when actively performing the task, pupil dilations have a dominating component of internal decision variables, while under passive performance external occurrences dominate the dilation and despite a lack of cognitive requirement to attend to the task, these passive behavioral states differ from complete disengagement (Figure 5B). These observations may relate to the LC adaptive gain theory and the exploration-exploitation modes (Aston-Jones et al., 1994, 1999; Usher et al., 1999; Clayton et al., 2004; Aston-Jones and Cohen, 2005). Hence, when the stimulus has no task function, but there exists a requirement to drive attention to external occurrences (such as potential reward) internal states quickly fluctuate from an exploitative mode, reflected by low baseline and pupillary reactivity to relevant occurrences, to an explorative mode where task occurrences are not coded, exhibited by increased baseline and low pupillary reactivity to the occurrence with baseline pupil size reflecting task attentiveness rather than mere arousal. Thus, the behavioral state and requirements posed by the environment are determining what the pupil reflects.

Taken together, our results provide further evidence for the complexity of what pupillary dilations reflect and support findings related to the LC-NE adaptive gain theory. When actively performing the task, these dilations provide evidence for a representation of response confidence. Also, baseline pupil size reflects states of task engagement and attentiveness rather than general arousal. Finally, in a state of passive behavior, pupillary dilations reflect external occurrences with smaller pupil dilation relative to active performance due to the lack of task-related decisional variables. The presented paradigm combined with pupillometry provides a framework to relate behavioral states with large scale neuronal network dynamics recorded using multielectrode (Jun et al., 2017) or two-photon imaging (Margolis et al., 2014; Stirman et al., 2016) techniques during perceptual decision making tasks.

## Data Availability Statement

The raw data supporting the conclusions of this article will be made available by the authors, without undue reservation.

## Ethics Statement

The animal study was reviewed and approved by North Rhein-Westphalia State Agency for Nature, Environment and Consumer Protection (Landesamt für Natur, Umwelt und Verbraucherschutz Nordrhein-Westfalen, LANUV).

## Author Contributions

DG, BK, and FH: contributed to the conception and designed the research. DG, MG, AB, and P-MG: performed research. DG and AB: analyzed the data. DG: wrote the first draft of the article. All authors contributed to the article and approved the submitted version.

## Conflict of Interest

The authors declare that the research was conducted in the absence of any commercial or financial relationships that could be construed as a potential conflict of interest.
